# Pacemakers and methylprednisolone pulse therapy in immune-related myocarditis concomitant with complete heart block

**DOI:** 10.1515/med-2022-0611

**Published:** 2022-12-20

**Authors:** Chunhong Hu, Lishu Zhao, Chengzhi Zhou, Hanping Wang, Shun Jiang, Yizheng Li, Yurong Peng, Chao Deng, Fang Ma, Yue Pan, Long Shu, Yan Huang, Yue Zeng, Fang Wu

**Affiliations:** Department of Oncology, The Second Xiangya Hospital, Central South University, Changsha, Hunan, China; State Key Laboratory of Respiratory Disease, National Clinical Research Center for Respiratory Disease, Guangzhou Institute of Respiratory Health, The First Affiliated Hospital of Guangzhou Medical University, Guangzhou, China; Department of Respiratory Medicine, Peking Union Medical College Hospital, Beijing, China

**Keywords:** immune checkpoint inhibitor, myocarditis, complete heart block, pacemaker, methylprednisolone pulse therapy

## Abstract

Immune-related cardiotoxicities are uncommon but potentially fatal. The study aims to evaluate the value of pacemakers and methylprednisolone pulse therapy (MPPT) to patients with immune-related myocarditis concomitant with complete heart block (CHB). We first reviewed medical records of three patients with immune-related myocarditis concomitant with CHB. For the pooled analysis, we searched related cases with immune-related myocarditis in the PubMed database and screened the patients. Clinical characteristics, management, and outcomes were summarized. Our three patients developed immune-related myocarditis concomitant with CHB about 2 weeks after receiving pembrolizumab, and were successfully treated with pacemaker implantation and high-dose steroids (two received MPPT). In the pooled analysis, 21 cases were eligible with an overall fatality rate of 52%. Patients with pacemakers had a fatality rate of 38%, significantly lower than patients without them (38% vs 100%; *p* = 0.035), particularly the MPPT subgroup (25% vs 100%; *p* = 0.019). All five patients without pacemakers expired. Among patients with pacemakers, MPPT patients tended to have an inferior rate compared with non-MPPT patients. Timely pacemaker implantation played a crucial role in improving the outcomes of patients with immune-related myocarditis concomitant with CHB. Patients receiving MPPT appeared to have a better prognosis. Additionally, multidisciplinary consultation should be recommended for better management.

## 1 Introduction

In recent years, immune checkpoint inhibitors (ICIs) have revolutionized antineoplastic protocols due to superior efficacy and better tolerance in comparison with traditional cancer therapies [1]. ICIs restore anti-tumor immune response of T lymphocytes by targeting PD-1, PD-L1, or CTLA-4, which may also contribute to systematic immune-related adverse effects (irAEs) [2]. Among various irAEs, immune-related cardiotoxicities are infrequent but increasingly reported [3–6], including myocarditis, pericarditis, arrhythmias, conduction abnormalities, and impaired ventricular function [7–10]. Despite a low incidence ranging from 0.06 to 1.1%, immune-related myocarditis has a high fatality rate of 20–65% [11–13]. Conduction abnormalities are regarded as the death mode of immune-related myocarditis [14]. Complete heart block (CHB), a form of conduction abnormalities, occurs in approximately 8% of patients with immune-related myocarditis and 64% of death cases, appearing to be particularly associated with mortality [15,16].

Immune-related cardiotoxicities brought unique challenges to clinicians due to heterogeneous symptoms, life-threatening characteristics, and the time-critical need to differentiate from non-ICIs-mediated cardiac dysfunction [10]. Some guidelines suggested permanently discontinuing ICIs and initiate high-dose corticosteroids (1–2 mg/kg/day), as well as transfer to coronary care units urgently for patients with myocarditis [17,18]. The National Comprehensive Cancer Network panel recommended transient pacemaker for patients with immune-related arrhythmia, and methylprednisolone pulse dosing (1 g/day for 3–5 days) for patients with ≥grade 3 immune-related myocarditis [14]. However, data supporting the use of pacemaker were limited to a single case report, and the role of pacemakers and methylprednisolone pulse therapy (MPPT; 500–1,000 mg/day for 3–5 days) in immune-related myocarditis accompanied by CHB has not been fully discussed.

Hence, we started by describing the successful management of three patients with immune-related myocarditis accompanied by CHB. All implanted the pacemaker and two received MPPT. To further evaluate the role of pacemaker implantation and MPPT, published cases plus three unpublished patients harboring immune-related myocarditis concomitant with CHB were concluded and compared in the pooled analysis.

## 2 Methods

### 2.1 Clinical data collection and literature search

All medical histories, test results, management, and outcomes of patients with myocarditis concomitant with CHB were obtained from the electronic medical record system of the three hospitals, which were the Second Xiangya Hospital of Central South University, the First Affiliated Hospital of Guangzhou Medical University, and Peking Union Medical College Hospital. All of them signed informed consent. Then the PubMed database was searched to retrieve cases diagnosed with immune-related myocarditis published in English by Feb 6, 2020, and screened patients accompanied by CHB. Clinical characteristics, management, and outcomes were concluded and compared between patients with and without a pacemaker.


**Ethics statement:** The study was approved by the ethics committee of the Second Xiangya Hospital, Central South University (No. 2020045). Written informed consent to participate and for publication was obtained from the patients.

### 2.2 Statistical analysis

Non-normally distributed age was expressed as median, interquartile range, and compared with the Mann–Whitney *U*-test. Categorical variables were described as numbers and percentages and compared with Fisher’s exact test. Statistical analyses were performed using SPSS version 22.0. It is statistically significant if two-sided *α* was less than 0.05.

## 3 Results

### 3.1 Case presentation

#### 3.1.1 Patient 1

**Figure 1:**
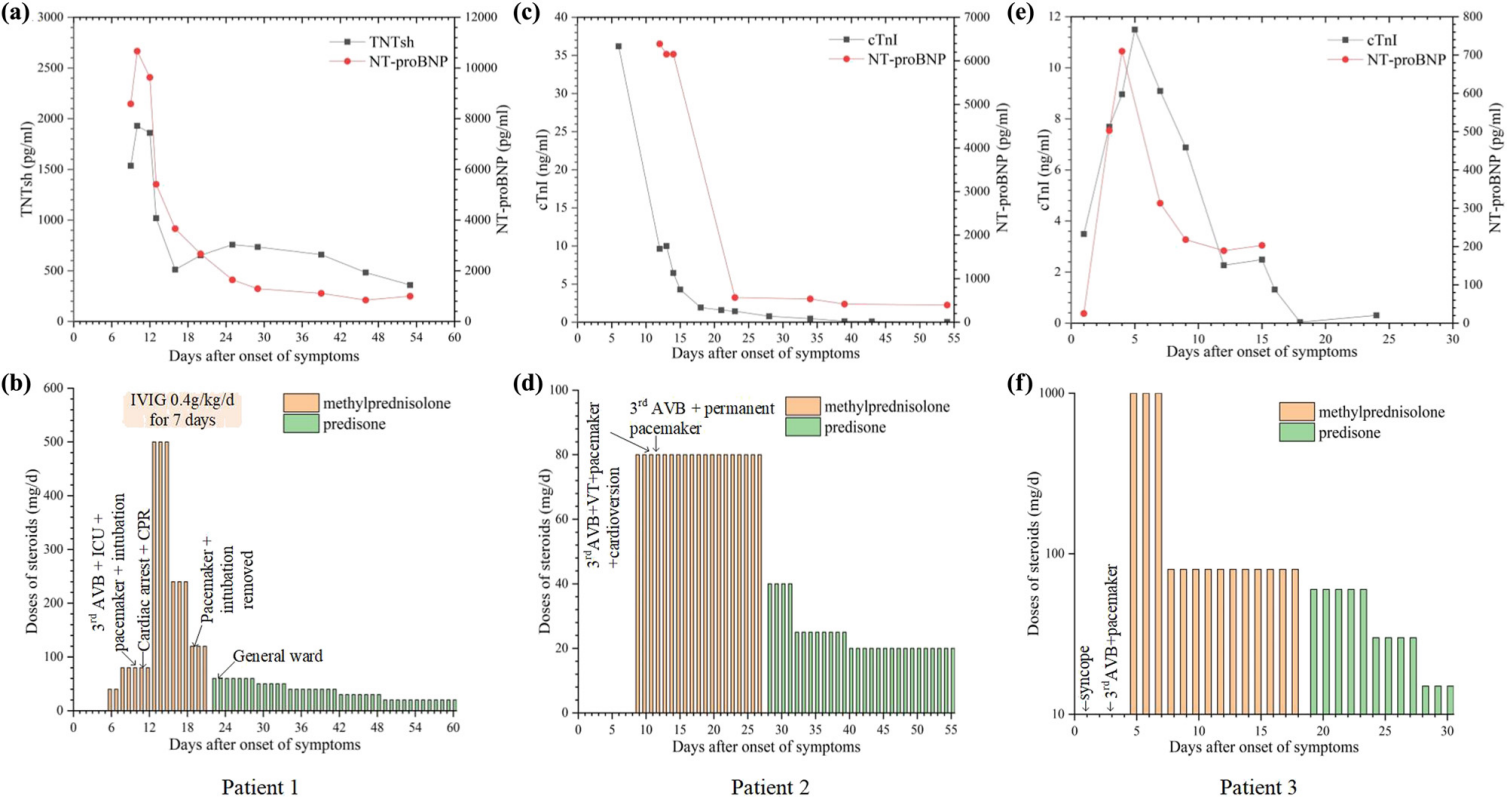
Clinical courses of three patients with immune-related myocarditis concomitant with complete AVB. (a, c, e) Cardiac biomarkers of three patients increased after developing myocarditis and gradually decreased with the initiation of therapy. (b, d, f) Doses of steroids over time and pacemaker implantation in three patients. TNTsh, high-sensitivity troponin T; NT-proBNP, n-terminal pro-brain natriuretic peptide; cTnI, cardiac troponin I; AVB, atrioventricular block; IVIG, intravenous immunoglobulin; and CPR, cardiopulmonary resuscitation.

A 70-year-old man with hypertension had small cell lung cancer. After two cycles of platinum-based chemotherapy with partial remission, he declined it due to severe gastrointestinal adverse reactions and urged immunotherapy. He presented with diplopia, myalgias of limbs, ophthalmalgia, and bilateral asymmetric ptosis 16 days after the first dose of pembrolizumab. Magnetic resonance imaging of eyes revealed bilateral symmetrically thickened extraocular muscles. Thyroid function tests and autoimmune serology were normal, but aspartate aminotransferase (AST) and alanine aminotransferase (ALT) elevated. The patient was given intravenous methylprednisolone at 40 mg/day due to possible immune-related adverse events. Then he developed choking and hoarseness, so methylprednisolone was increased to 80 mg/day. After neurology consultation, he was transferred to the neurology ward and received intravenous immunoglobulin 400 mg/kg/day. Within 12 h, the patient presented with dysphagia, aconuresis, dyspnea, and drowsiness. The electrocardiogram (ECG) monitoring prompted sinus bradycardia with heart rates of 30–50 bpm, atrial bigeminy, and ST-segment elevation, soon deteriorating to third-degree atrioventricular block. High-sensitivity troponin T and NT-proBNP rose to 1,536 and 8,583 pg/mL, respectively. He was transferred to the intensive care unit (ICU). Cardiac biomarkers revealed cTnT 1,930 pg/mL, creatine kinase (CK) 2,304 U/L, creatine kinase isoenzyme (CK-MB) 138 U/L, and NT-proBNP 10,663 pg/mL. Meanwhile, the concentration of blood calcium was 1.95 mmol/L and the concentration of serum sodium was 135.1 mmol/L, which were all lower compared to the normal baseline values (before the immunotherapy). Echocardiograms revealed a normal left ventricular ejection fraction (LVEF) of 59%. Coronary angiography showed a 70% stenosis of the diagonal branch and 75% stenosis of the posterior descending branch. He was intubated and had a temporary pacemaker implanted because of respiratory failure and third-degree atrioventricular block. On the second day in ICU, ventricular tachycardia attacked, then deteriorating to cardiac arrest. Fortunately, he revived after cardiopulmonary resuscitation. After multidisciplinary consultation, immune-related myositis and myocarditis were clinically diagnosed. And we started methylprednisolone 500 mg/day for 3 days, then weaned half dose every 3 days. After a week, the patient gradually recovered, and the cardiac biomarkers and transaminase had an apparent decline, so we removed mechanical ventilation and the temporary pacemaker, and commenced prednisone initially 60 mg/day weaned by 10 mg every week (Figure 1a and b). Four months after developing myocarditis, positron emission tomography-computed tomography showed partial remission, and he underwent two doses of chemotherapy and progressed until 1 year later.

#### 3.1.2 Patient 2

A 67-year-old male who received one dose of pembrolizumab combined with platinum-based chemotherapy for large cell neuroendocrine carcinoma presented with palpitations, chest discomfort, limb weakness, systemic rashes, bilateral ptosis, and blurred vision 14 days later. Initial laboratory results were notable for elevated cardiac enzymes (CK 11,537 U/L, CK-MB 254 U/L, cTnI 36.2 ng/mL; Figure 1c) and transaminase (ALT 294 U/L, AST 679 U/L). ECG showed ST-T segment abnormalities. The echocardiogram revealed a normal LVEF (62%), and coronary angiography contradicted coronary artery disease. Based on these, immune-related myocarditis and myositis were clinically diagnosed and finally confirmed by muscle biopsy that revealed focal lymphocytes infiltrated in the striated muscles. He was given intravenous methylprednisolone 80 mg/day and intravenous immunoglobulin 10 g for 4 days. Despite these measures, frequent second- or third-degree atrioventricular block and ventricular tachycardia took place, and subsequently, he had a temporary pacemaker implanted. Soon after this, he was transferred to a state of confusion and underwent electric defibrillation because of ventricular tachycardia. On account of persistent third-degree atrioventricular block, a permanent pacemaker was placed. Intravenous methylprednisolone 80 mg/day continued for over half a month followed by slowly weaned prednisone initially 40 mg/day (Figure 1d). With the above treatment for over 1 month, he was discharged due to substantial improvements in cardiac biomarkers and symptoms (Figure 1c).

#### 3.1.3 Patient 3

A 57-year-old man harbored advanced lung adenocarcinoma and presented with recurrent syncope 14 days after two doses of pembrolizumab combined with chemotherapy. His medical history included hypertension and diabetes mellitus. ECG revealed a second-degree atrioventricular block. Initial workup prompted myocarditis (CK 2,784 U/L, CK-MB 89 U/L, cTnI 3.49 ng/mL, NT-proBNP <50 pg/mL; Figure 1e). Echocardiogram and coronary angiography were unremarkable. On the third day of presentation, cardiac biomarkers were higher than before (CK 3,130 U/L, CK-MB 53.1 µg/L, cTnI 7.69 ng/mL, NT-proBNP 503 pg/mL). ECG showed a third-degree atrioventricular block, and a temporary pacemaker was immediately implanted. On the fifth day, the patient received methylprednisolone intravenously 1 g/day for 3 days followed by 80 mg/day. Repeat cTnI and NT-proBNP showed a gradual decline (Figure 1e). Oral prednisone commenced initial 60 mg/day then slowly weaned and ceased over 6 weeks (Figure 1f).

### 3.2 The pooled analysis

Three patients as above and 18 published cases with immune-related myocarditis were eligible for the pooled analysis [3,5,19–34]. We concluded the clinical characteristics and outcomes of 21 patients, including 16 with pacemakers and five without it (Table 1). The median age was 66 years, and males accounted for 62%. Thirty-three percent of patients had previous hypertensive disease. The most common indications for ICIs were melanoma and lung cancers with nivolumab and pembrolizumab being predominant ICI types. Nineteen (90%) received only 1–2 cycles of immunotherapy at the time of onset with nonspecific myocarditis symptoms. Concurrent toxicity, most commonly myositis, accounted for more than half of the myocarditis cases. Ninety percent had troponin elevation and 62% had preserved LVEF. All patients received steroids therapy, and 16 (76%) were MPPT users.

**Table 1:** Characteristics of patients with immune-related myocarditis concomitant with CHB

	All patients (*n* = 21)	With pacemaker (*n* = 16)	Without pacemaker (*n* = 5)	*p-*Value
Male, *n* (%)	13 (62)	11 (69)	2 (40)	0.325
Age, median (IQR), y	66 (63–70)	66 (62–72)	66 (55–67)	0.660
Comorbidities, *n* (%)				
Hypertension	7 (33)	6 (38)	1 (20)	0.624
Hypercholesterolemia	4 (19)	3 (19)	1 (2)	0.998
Diabetes	3 (14)	3 (19)	0 (0)	0.579
Cancer types				
Melanoma, *n* (%)	5 (24)	3 (19)	2 (40)	0.546
Lung cancer, *n* (%)	9 (43)	8 (50)	1 (20)	
Others, *n* (%)	7 (33)	5 (31)	2 (40)	
Unknown				
ICIs, *n* (%)				
Nivolumab	9 (43)	8 (50)	1 (20)	0.546
Pembrolizumab	7 (33)	5 (31)	2 (40)	
Others	5 (24)	3 (19)	2 (40)	
Onset after 1–2 doses of ICIs, *n* (%)	19 (90)	14 (88)	5 (100)	1.000
Symptoms, *n* (%)				
Fatigue or muscle weakness	13 (62)	10 (63)	3 (60)	1.000
Dyspnea	9 (43)	5 (31)	4 (80)	0.119
Myalgias	6 (29)	5 (31)	1 (20)	1.000
Ocular signs	5 (24)	4 (25)	1 (20)	1.000
Elevated troponin, *n* (%)	19 (90)	14 (88)	5 (100)	1.000
LVEF <50, *n* (%)	7 (38)	5 (31)	2 (40)	1.000
Coronary angiography, *n* (%)				
Normal	10 (48)	8 (50)	2 (40)	0.823
Minor stenosis	5 (24)	4 (25)	1 (20)	
Unknown	6 (28)	4 (25)	2 (40)	
Biopsy or autopsy, *n* (%)	13 (62)	9 (56)	4 (80)	0.606
Treatment, *n* (%)				
Corticosteroid	21 (100)	16 (100)	5 (100)	N/A
MPPT	16 (76)	12 (75)	4 (80)	1.000
IVIG	4 (19)	3 (19)	1 (20)	1.000
Deaths, *n* (%)	11 (52)	6 (38)	5 (100)	**0.035**
Cause of death, *n* (%)				
Cardiovascular events	6 (29)	4 (25)	2 (40)	0.598
Abandoning treatment	5 (24)	2 (18)	3 (60)	N/A
Die of cardiovascular events, *n* (%)				
Lethal arrhythmia	1 (17)	1 (25)	0 (0)	N/A
Heart failure	3 (50)	2 (50)	1 (50)	
Cardiogenic shock	2 (33)	1 (25)	1 (50)	
MPPT, *n* (%)	16 (76)	12 (75)	4 (80)	N/A
Deaths, *n* (%)	7 (44)	3 (25)	4 (100)	**0.019**
Non-MPPT, *n* (%)	5 (24)	4 (25)	1 (20)	N/A
Deaths, *n* (%)	4 (80)	3 (75)	1 (100)	1.000

Abbreviations: IQR, interquartile range; ICIs, immune checkpoint inhibitors; LVEF, left ventricular ejection fraction; MPPT, methylprednisolone pulse therapy; IVIG, intravenous immunoglobulin; and N/A, not applicable.

There was no significant difference in clinical characteristics and treatment between patients with pacemakers or not. The overall fatality rate was 52% (11 of 21). The fatality rate of patients with a pacemaker was significantly lower than that of patients without it (38% [6 of 16] vs 100% [5 of 5]; *p* = 0.035), especially in the MPPT subgroup (25% [3 of 12] vs 100% [4 of 4]; *p* = 0.019). Additionally, all five patients without a pacemaker died whether they received MPPT or not. Among patients with pacemakers, MPPT patients seemed to have a lower fatality rate compared with non-MPPT patients despite no statistical significance (25% [3 of 12] vs 75% [3 of 4]; *p* = 0.118). Moreover, ten patients received MPPT as the initial therapy, and seven of them survived.

## 4 Discussion

In the era of immunotherapy for cancers, immune-related myocarditis is infrequent but has a high mortality, which was associated with conduction abnormalities, particularly CHB [35]. We performed the study because not enough evidence-based data supported the application of the pacemaker and MPPT in immune-related myocarditis with CHB. First, we reported three patients who suffered early-onset immune-related myocarditis, and all had pacemakers placed on the same day that CHB occurred. Two of them received MPPT and then all recovered with a rapid decline of troponin, a negative prognostic predictor for immune-related myocarditis [16,36]. Therefore, we suspect that timely pacemaker implantation and high-dose steroids, especially MPPT, may forestall deaths caused by immune-related myocarditis concomitant with CHB, which is further demonstrated in the pooled analysis.

Among all 21 patients, one-third were not confirmed with an endomyocardial biopsy (EMB) or autopsy that is the golden diagnostic criteria for immune-related myocarditis because EMB is invasive and hard to perform in routine clinical practice. Nonetheless, other patients can be diagnosed with clinically suspected myocarditis if there were ≥1 clinical presentation (such as dyspnea and fatigue) and ≥1 diagnostic criterion (ECG abnormalities, troponin elevation, abnormalities on echo/angio/CMR, and CMR tissue abnormalities) [37]. The mechanism of immune-related myocarditis, though not clearly clarified, is related to the infiltration of predominant CD4 and CD8 positive T lymphocytes and a few macrophages (CD68+ cells) in the myocardium [3,38]. Moreover, shared antigens and similar T cell clones have been found between tumors and cardiac muscles [3]. Under this circumstance, T lymphocytes and macrophages infiltrate the myocardium following the initiation of ICIs, thereby inducing immune-related myocarditis. In our study, most patients presented symptoms after 1–2 cycles of immunotherapy and had a high fatality rate of up to 52%, in accordance with previous studies [11]. The symptoms were diverse, such as dyspnea, fatigue, and chest pain, but more than half of the patients harbored simultaneous myositis manifesting as myalgias and ocular signs, tending to be more frequent than previously reported (25%) [11]. It may be caused by shared antigens presenting in the tumors, myocardium, and striated muscle [3].

A majority of patients with immune-related myocarditis had a preserved LVEF even after the development of CHB, which indicates clinicians should not regard LVEF as a discriminator of severe myocarditis. On the contrary, over 90% of patients had troponin elevation, and troponin T ≥1.5 ng/mL predicted an increased risk of major adverse cardiac events [16]. Given that the median time from ICIs initiation to myocarditis was 34 days [16], it is of great importance to detect troponin levels before and after ICIs therapy, particularly after 1–2 doses of ICIs.

CHB often warranted intervention and even constituted indications for implanting temporary or permanent pacemaker [39]. However, in our study, five patients did not implant the pacemaker in the presence of CHB induced by ICIs, and all of them passed away. Two of them refused to implant the pacemaker [5,34], while three were not recommended by their doctors [31–33], which suggested the necessity of consulting cardiologists for pacemaker implantation did not raise enough attention for clinicians, particularly oncologists. In contrast to patients without a pacemaker, those with it had a lower overall fatality rate, particularly in the MPPT subgroup. It prompts that timely pacemaker implantation may play a critical role in improving the outcomes of patients harboring immune-related myocarditis concomitant with CHB. In the clinical setting, multidisciplinary collaboration, especially in consultation with cardiologists should be recommended for such patients to promote early diagnosis and intervention.

Steroids were widely applied in immune-related myocarditis to suppress lymphocyte activity and inhibit cytokine synthesis, but the treatment timing and doses remained non-uniform. Evidence showed higher steroid doses (such as 1–2 mg/kg of prednisone) were associated with a lower rate of major adverse cardiovascular events [16]. However, high-dose steroids may not be sufficient to reverse cardiac toxicity [3,40]. Methylprednisolone 1 g/day intravenously was recommended for patients without an immediate response to high-dose steroids, and as the initial therapy for patients with grade 3 or 4 immune-related myocarditis [18,14]. Our most intriguing result was that 12 MPPT patients with pacemakers tended to have a lower fatality rate than non-MPPT patients. Despite no statistical significance, it lays foundations for future large-scale studies and better management of severe or life-threatening immune-related cardiac toxicities. Although to date, we only analyzed ten patients receiving MPPT as initial treatment, we believe that these results may represent a paradigm underlying MPPT as the initial therapy, which might be a new model of steroid therapy for severe or life-threatening myocarditis. Ten patients received MPPT as the initial therapy, which might be a new model of steroid therapy for severe or life-threatening myocarditis. Moreover, if no obvious improvement, other immunosuppressive drugs should be added, such as intravenous immunoglobulin and anti-thymocyte globulin [14].

## 5 Conclusion

Timely pacemaker implantation played a crucial role in improving the outcomes of patients with immune-related myocarditis concomitant with CHB. Patients receiving MPPT appeared to have a better prognosis. Additionally, multidisciplinary consultation should be recommended for better management of immune-related cardiotoxicities.
